# High-Performance Temperature Sensor by Employing Screen Printing Technology

**DOI:** 10.3390/mi12080924

**Published:** 2021-08-02

**Authors:** Zhaojun Liu, Bian Tian, Bingfei Zhang, Zhongkai Zhang, Jiangjiang Liu, Libo Zhao, Peng Shi, Qijing Lin, Zhuangde Jiang

**Affiliations:** State Key Laboratory for Mechanical Manufacturing Systems Engineering, Xi’an Jiaotong University, Xi’an 710000, China; bingfeizhang01@stu.xjtu.edu.cn (B.Z.); zhangzk@xjtu.edu.cn (Z.Z.); super_l@stu.xjtu.edu.cn (J.L.); libozhao@mail.xjtu.edu.cn (L.Z.); spxjy@mail.xjtu.edu.cn (P.S.); qjlin2015@xjtu.edu.cn (Q.L.); zdjiang@mail.xjtu.edu.cn (Z.J.)

**Keywords:** temperature sensor, screen printed, indium oxide, sensitivity

## Abstract

In the present study, a high-performance n-type temperature sensor was developed by a new and facile synthesis approach, which could apply to ambient temperature applications. As impacted by the low sintering temperature of flexible polyimide substrates, a screen printing technology-based method to prepare thermoelectric materials and a low-temperature heat treatment process applying to polymer substrates were proposed and achieved. By regulating the preparation parameters of the high-performance n-type indium oxide material, the optimal proportioning method and the post-treatment process method were developed. The sensors based on thermoelectric effects exhibited a sensitivity of 162.5 μV/°C, as well as a wide range of temperature measurement from ambient temperature to 223.6 °C. Furthermore, it is expected to conduct temperature monitoring in different scenarios through a sensor prepared in masks and mechanical hands, laying a foundation for the large-scale manufacturing and widespread application of flexible electronic skin and devices.

## 1. Introduction

As electronic products are being developed to be increasingly small, light and thin, flexible printed circuit boards (FPCB) have been extensively applied in several fields (flexible displays and flexible sensors) for their small size, light weight, static bending and dynamic folding and curling [1,2,3,4]. Conductive ink, as a vital material of FPCB, has aroused huge attention in electronic technology [5,6]. On the whole, conventional conductive inks consist of binders, conductive materials, solvents and relevant additives. To be specific, the conductive materials generally refer to carbon materials (e.g., graphite, carbon nanotubes and conductive carbon black), or metal particles (e.g., silver and copper) [7,8,9,10]. However, as flexible electronic devices are being increasingly demanded, and the manufacturing technology is leaping forward, numerous materials are continuously emerging, which can undergo a close integration with flexible polymer substrates via a multi-style manufacturing process, as an attempt to replace conventional conductive inks. Screen printing technology refers to a film preparation method that is characterized by simple technology, low cost and easy mass production. By integrating different printing pastes and supporting objects, various functional films can be prepared, which lays the basis for developing high-performance sensors or devices [11,12,13].

As impacted by the mentioned advantages, screen printed technology turns out to be one of the hotspot technologies for film preparation. In 2015, Zhuo Cao et al. presented the fabrication and testing of bismuth tellurium-antimony tellurium-based flexible thermocouples by employing screen printing technology for energy harvesting application. The Seebeck coefficient of the thermocouple printed on a flexible polyimide substrate ranges from 193 to 227 μV/K, so the low-cost screen printing technology and low-temperature curing materials are demonstrated to be promising for fabricating flexible thermoelectric generators [14]. In 2018, based on screen printing, Liu et al. prepared an indium oxide (In_2_O_3_)/indium tin oxide (ITO) thin film thermocouple to achieve high-temperature measurements. Through the optimization of the mass fraction of binder, a compact temperature sensor with a sensitivity of 44.5 μV/K has been developed [15]. In 2020, Md et al. employed screen printing to fabricate a thermoelectric functional material that was characterized by high repeatability, reproducibility, stability and great application potential, and had the thermoelectric power factor over 500 μW/m·K^2^. Based on Ag-Se-based materials, thirteen thermocouples composed of this material on the polyethylene glycol terephthalate were employed to fabricate a flexible folding thermoelectric generator [16].

Accurate temperature measurements are capable of efficiently addressing numerous critical problems and presenting vital information [17,18,19,20,21]. In the present study, a screen printing process that applies to polyimide was proposed to fabricate high-performance temperature-sensitive films. The n-type semiconductors In_2_O_3_ and ITO that exhibited prominent thermoelectric properties were selected as the sensitive layer materials to achieve temperature sensing, which were expected to constitute a high sensitivity thermocouple temperature sensor [15,22]. Given the limitation of the low sintering temperature of flexible polymer substrates, a method by exploiting screen printing technology and a low-temperature heat treatment process suitable for polymer substrates were proposed and achieved. The fabricated sensor was suggested to exhibit excellent performance, which could identify a slight variation in temperature and undergo the sensitivity test effectively. Moreover, the sensor demonstrated high-performance temperature detection capability in manipulator and intelligent wearable device manufacturers.

## 2. Fabrication

**Figure 1:**
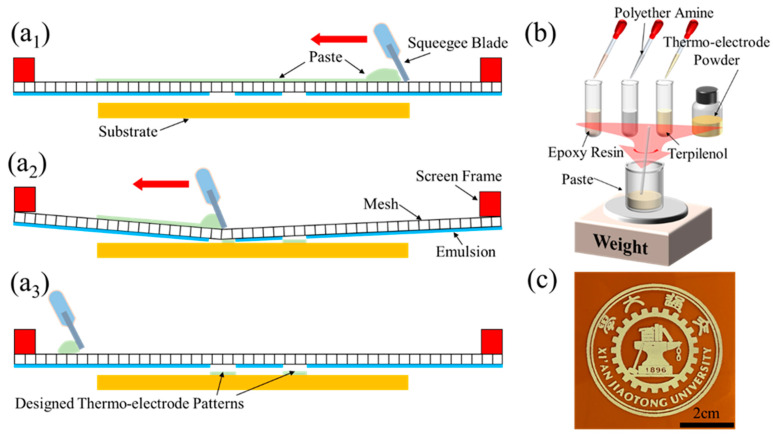
Illustration of pattern deposition by applying screen printing technology: (**a**) Flowchart of printing a layer of thermo-electrode on the flexible polyimide substrate. The slurry was proportionally prepared indium oxide/indium tin oxide powder; (**b**) Slurry preparation. Here, epoxy resin was a bisphenol-A type resin with an average epoxy value of 0.51. Polyether amine (D-400) was a type of polymer with the main chain of polyether structure. The terminal active functional groups in the structure were amine groups. The molecular weight was 400; (**c**) Design patterns prepared by applying screen printing technology.

Epoxy resin (E-51) and polyether amine (D-400) were taken as the binder and curing agent to bond the thermo-electrode powder In_2_O_3_ and ITO. In addition, α-Terpineol was selected as the thermal electrode powder solvent and curing catalyst [23,24,25]. The polyimide that exhibited higher glass transition temperature was selected as the flexible substrate of the sensor. Moreover, a wide range of materials were proportioned in accordance with a certain mass ratio to produce the slurry to be applied in the screen printing process. Figure 1a depicts the schematic diagram of the screen printing process. In the experiment, the polyimide substrate was only below the pattern of the printed screen. Subsequently, the self-configured paste was squeezed via the grids in the middle of the customized screen with a scraper. In Figure 1b, epoxy resin, polyether amine, thermoelectric powder material and terpinol were placed in a disposable beaker by mass ratio and then overall mixed. In practice, to ensure the quality of the electrode, the electrode should be commonly brushed for several times to ensure the continuity of the thermo-electrodes. However, repeated brushing will cause the film thickness to increase, so the film will be broken and peeled off as impacted by thermal stress in the subsequent heat treatment. Furthermore, there is also a problem of weak bonding force between layers, which can cause the electrode to fall off. Thus, per thermo-electrode commonly conducts the process in Figure 1a once or twice. After each brushing, thermo-electrode was processed on a heating stage at 150 °C for nearly 15 min to ensure its complete solidification. Figure 1c illustrates a complex pattern prepared with screen printing technology. After the positive and negative thermal electrodes were prepared by screen printing in turn, the sensor was heat-treated at 350 °C to conduct thermal activation of the electrode materials.

To guide the practical curing process of epoxy resin and polyether amine, differential scanning calorimetry (DSC) and thermogravimetry analysis (TGA) were adopted to investigate the curing process of the system. The curing reaction was an addition reaction in which the amino groups on the polyether amine attacked the carbon molecules on the epoxy ternary of the epoxy resin to open the ternary ring. The reaction equation is expressed in Equation (1).

(1)
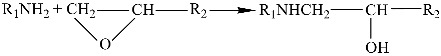


**Figure 2:**
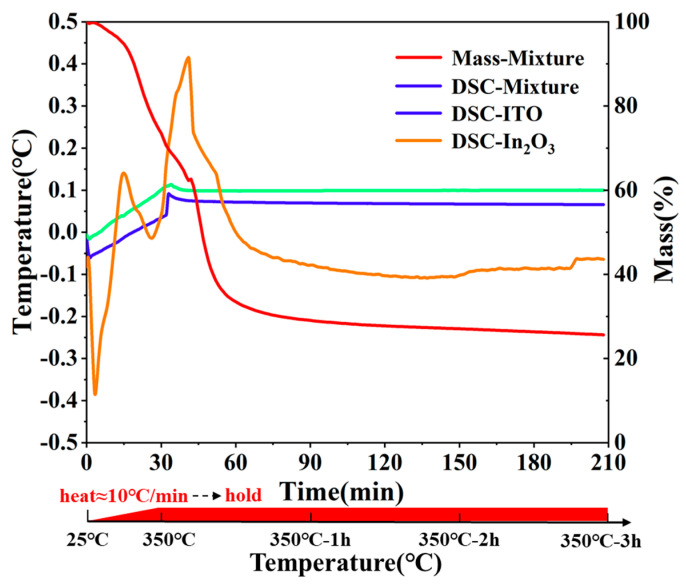
The DSC and TGA results of In_2_O_3_ powder, ITO powder and mixture (epoxy resin, polyether amine and terpinol).

Besides, the reaction should be heated to a certain temperature to boost the progress. Moreover, terpineol acted as a proton donor to catalyze the curing reaction. Given the limitation of the glass transition temperature of the flexible substrate, the maximum heating temperature could not exceed 350 °C. The DSC and TGA results are presented in Figure 2. The temperature was set to load two phases. In the air atmosphere, the temperature rose from ambient temperature to 350 °C in the first 30 min. The second stage was to keep the temperature at 350 °C for 3 h. In the entire heating process, the heat exchange and quality change data of the powder and mixture (50.0 wt.% epoxy resin, 27.3 wt.% polyether amine and 22.7 wt.% terpinol) were collected. As indicated from the heat flow curves of the In_2_O_3_ powder, ITO powder, two powders only absorbed a small amount of heat at the heating stage and the state remained stable during the 3 h heat preservation. By using this method, it could be proved that this low-temperature heat treatment exerts no effect on the thermo-electrode powder material, which can ensure the feasibility of the preparation. The large changes in the curve during the temperature phase change were attributed to the baseline drift of the equipment. For the results of the mixture, the mass of the mixture decreased significantly at the heating stage, whereas the decrease rate decreased after the temperature was maintained at 350 °C. Moreover, the DSC curve first showed two obvious exothermic peaks (50 –70 °C and 250 –280 °C) at the heating stage, which corresponded to the stage of addition reaction between epoxy group and polyether amine, as well as the stage of crosslinking polymerization of the additive, respectively. Two obvious endothermic peaks appeared simultaneously, which belonged to the volatilization of the solvent terpineol and the carbonization of the mixture. Since the boiling point of α-terpineol was 224 °C, α-terpineol was vaporized in the heating section. The solidified mixture tended to be pyrolyzed to form carbon and carbon oxides. To be specific, the solid product of pyrolysis could act as a binder of thermo-electrode powder to maintain the integrity and connectivity of the film by using screen printing technology. As impacted by the presence of residual binder between the powders, the tunneling effect was imposed on the carriers of the thermoelectric material during the directional movement.

The tunneling effect refers to a quantum effect determined by the volatility of microscopic particles, i.e., barrier penetration. The tunneling effect is to form a certain current path between the thermo-electrode powder particles. If the directional movement of the carriers in material powder is hindered by the temperature difference, such hindrance can be considered a potential barrier with a certain potential energy. In terms of quantum mechanics, even if the energy of a microscopic particle is less than that of the potential barrier, besides being probably reflected, the particle may pass through the barrier. The mentioned phenomenon is termed as penetration effect. The majority carriers of In_2_O_3_ and ITO are electrons that may pass through the barrier between conductive particles. The probability of electrons that pass through the isolation layer is associated with the thickness of the isolation layer, as well as the difference between the energy of the isolation layer and the energy of the electrons. The smaller the thickness and difference, the more probable the electrons will pass through the isolation layer. With the thickness of the isolation layer decreasing to a certain value, electrons are capable of easily passing through this thin isolation layer, and the isolation layer between the thermo-electrode powder particles acts as a conductive layer.

Accordingly, to prepare thermoelectric films by using screen printing, the ratio of materials and the selection of subsequent heat treatment processes are of high significance and can directly affect the practical performance exhibited by the temperature sensor. In the flexible temperature sensor comprising In_2_O_3_ and ITO, In_2_O_3_ acted as the main material dominating the sensitivity of the sensor. A range of thermoelectric films were fabricated by altering the mass ratio of In_2_O_3_ powder and binder, as an attempt to obtain a high-performance temperature sensor. In this study, epoxy resin and polyether amine were configured in equal mass ratio to ensure the consistency of curing degree. Furthermore, the In_2_O_3_ powder and the solvent terpineol maintained the identical mass ratio. The preparation parameters of slurries are listed in Table 1.

## 3. Characterization and Detection

**Figure 3:**
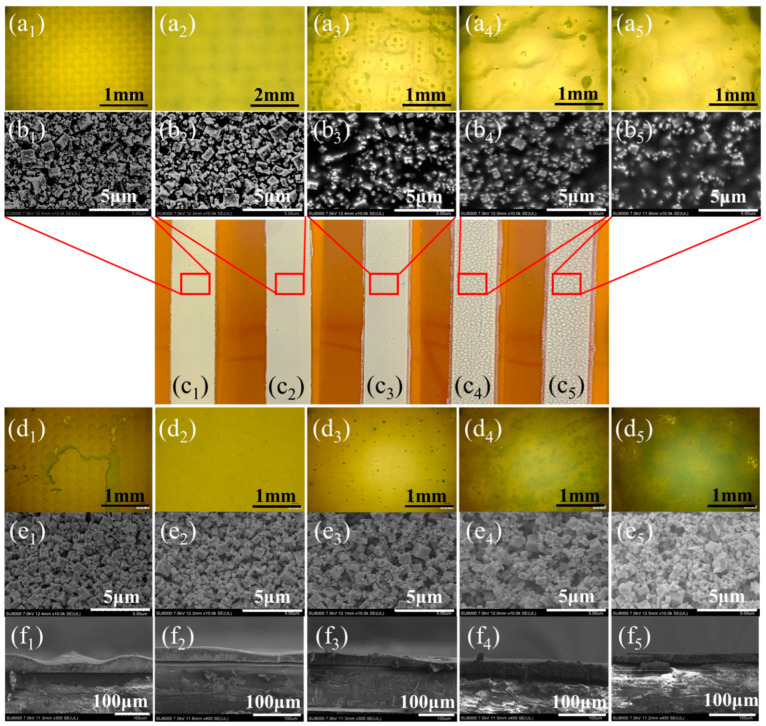
The optical and SEM images under different preparation parameters: (**a**) Morphology of groups 1–5 samples cured at 150 °C for 15 min after printing twice; (**b**) The SEM images of 1–5 samples before the heat treatment; (**c**) Macroscopic images of 5 groups of samples fabricated on the flexible substrates; (**d**) Morphology of groups 1–5 samples cured at 35 °C for 60 min; (**e**) The SEM images of 1–5 samples after the heat treatment; (**f**) The SEM images of the cross-section view of 1–5 samples.

According to Figure 3a–c, the cured films prepared by applying screen printing technology under different material ratios exhibited the completely different physical properties. First, as revealed from the macro picture comparison in Figure 3c, with the improvement of binder mass fraction, the surface of the film tended to be converted from a dense one to ‘arroyo’. According to the results achieved under the optical microscope (Figure 3a), the film surface of the first sample exhibited the identical grid-like structure to the screen. The In_2_O_3_ powder in the slurry took up a large mass fraction, so the prepared film lost the leveling property, which caused the surface of film to be ‘hill’. With the increase in the binder mass fraction, the film morphology tended to be flattened, whereas too excessive binder caused the powder to be completely wrapped, and the film exhibited a “hill” like morphology. As the mass fraction of the binder increased, the morphology of the film gradually was flat. However, excessive binder also caused the In_2_O_3_ powder to be overall coated, so a “volcano” appearance was created. The results achieved under scanning electron microscope (SEM) (Figure 3c) clearly indicated that with the improvement of the binder mass fraction, in the field of view, the morphology of the initial large-area block powder tended to be the solidified state of the resin. Figure 3d,e present the optical and SEM images of the samples after the samples were treated in the air at 350 °C for 1 h. The low content of binder resulted in poor film-base adhesion of the sample, which caused serious shedding of the film in Figure 3d_1_. As opposed to the mentioned, the excessive content of the binder caused large-area carbonization in Figure 3d_4_,d_5_ and considerable hole defects in Figure 3e_3_–e_5_ after the heat treatment of the film. Thus, it was obviously confirmed that an inappropriate slurry will directly affect the characteristics of the film and the performance of the sensor. Figure 3f shows the cross-section of each sample under SEM. It can be found that with the increase of binder mass fraction in the slurry, the viscosity (the viscosities of the five sample are 423,485 mPa·s, 102,755 mPa·s, 17,423 mPa·s, 4325 mPa·s and 3385 mPa·s, respectively) of the slurry decreases gradually, so that the uniformity and fluidity of the films are gradually improved. However, after the heat treatment, the Group 2 in Table 1 exhibited the advantages of a smoother surface and a denser film. Accordingly, the further performance test of the thermo-electrode prepared under this parameter was performed.

**Figure 4:**
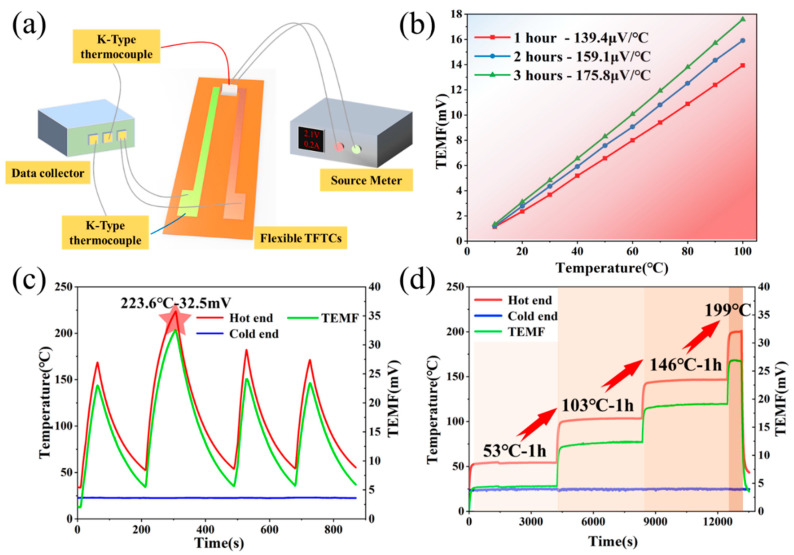
Comprehensive evaluation of sensor performance: (**a**) Self-designed calibration test system of sensor; (**b**) Comparison of the thermoelectric characteristics test of the different thermocouples (In_2_O_3_/Cu) (In_2_O_3_ thermo-electrode under different heat treatment periods). The optimized sensitivity of In_2_O_3_ thermo-electrode was 175.8 μV/°C; (**c**) Evaluation of test repeatability of sensors prepared by using screen printing technology (In_2_O_3_/ITO). The highest instantaneous temperature measured could be 223.6 °C; (**d**) The temperature retention of the sensor that was tested at different temperatures. The heat preservation test was performed at 53 °C, 103 °C and 146 °C, respectively, for up to 1 h. Besides, a short-term test of 10 min was performed at around 199 °C.

As indicated from the mass-mixture curve in Figure 2, a small decrease remained in the mixture quality at 350 °C continuous heat preservation. Thus, the thermoelectric properties of samples with different heat treatment times should be evaluated to determine the optimal thermoelectric properties of the thermoelectric film. To perform the calibration test of the flexible temperature sensor in the low temperature section, a dedicated experimental platform (Figure 4a) was built. To ensure the identical test environment for different thermo-electrodes, the samples after different heat treatments were adhered onto the copper tape to form the temperature-sensitive end of the thermocouples (In_2_O_3_/Cu) at the adhesive. In such a type of thermocouples, the Seebeck coefficient of copper as the reference electrode nearly reached 0.5 μV/°C, which could be negligible compared with In_2_O_3_ thermo-electrode. The thermal resistance by employing the source meter as the power supply device was placed on the temperature sensitive part of the thermocouples as a temperature stimulus, and a standard commercial temperature sensor was selected to monitor the temperature of the hot and cold ends of the thermocouples. Moreover, a multi-channel digital multimeter (Keithley DAQ6510) was employed to collect the thermal electromotive force (TEMF) and the temperature data in real time. By altering the heat source temperature to form a temperature difference between the hot and cold ends of the thermocouple, the TEMF of the thermocouples with different heat treatment periods at a temperature difference of 0–100 °C was determined, respectively. As suggested from the comparison of the three sets of thermocouples in Figure 4b, during the heat treatment, the In_2_O_3_ film exhibited higher test performance. After 3 h of heat treatment, the sensitivity could reach up to 175.8 μV/°C. After the optimal material ratio and post-processing process parameters were determined, screen printing technology was adopted to prepare a flexible temperature sensor that was composed of In_2_O_3_ and ITO. By exploiting the self-built test system, the prepared samples underwent the repeated temperature rise and the fall cycle tests (Figure 4c). The highest instantaneous temperature of sensor was 223.6 °C, the TEMF value could reach 32.5 mV, while the test sensitivity was 162.5 μV/°C. As ensured by the ultra-high sensitivity, the sensor could recognize slight temperature changes. The prepared high-performance sensors have outperformed human skin in temperature measurement range and sensitivity to temperature. The fabricated sensor could detect temperature changes indistinguishable to humans or satisfy the temperature measurement requirements in extreme endurance environments. Besides, the temperature retention of the sensor also acted as a key factor in the actual test. Four-stage temperature retention was randomly selected for the sensor to evaluate the temperature retention. The entire test was performed for nearly 4 h (Figure 4d). At the test temperature of 53 °C, 103 °C and 146 °C, the temperature drift rate of the sensor reached 3.21 °C/h, 6.87 °C/h and 5.18 °C/h, respectively, which ensured the measurement accuracy and stability of the sensor during service. Lastly, the sensor could still maintain great working stability and reliability under the thermal excitation of 199 °C.

## 4. Experiment and Test

**Figure 5:**
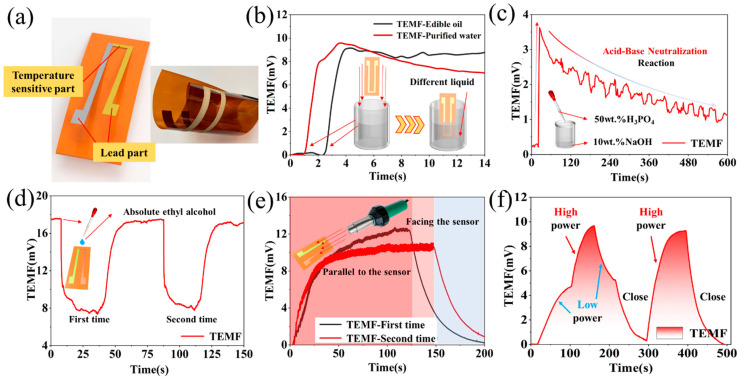
Conduct a full range of practical application tests on the sensor. Perform multiple tests on the sensor in the actual application environment and different temperature stimuli: (**a**) Schematic diagram and the sample of the flexible temperature sensor; (**b**) Temperature test of hot soybean oil and deionized water solution; (**c**) Monitor the real-time changes in the temperature of the acid-base neutralization reaction; (**d**) Temperature measurement of absolute ethyl alcohol volatilization; (**e**) Airflow temperature detection of hot air welding machine; (**f**) Temperature detection of electric heaters based on heat radiation.

The prepared temperature sensor that exhibited great service stability and reliability was suggested to show wide application prospects and value. The flexible sensor underwent a full range of application testing. On the one hand, the temperatures of the solid, liquid and gaseous substances to be tested were detected, respectively. On the other hand, the sensor conducted the temperature measurement of different heat transfer methods (e.g., convective heat transfer, solid heat transfer, and thermal radiation). In Figure 4b,c, the thermal resistance was employed as the temperature source to measure the solid heat transfer temperature of the sensor. Furthermore, the temperature of the hot air flow and the hot liquid was measured to determine the performance of the sensor in Figure 5a–f.

In Figure 5a,b, the flexible sensor attached onto the robotic finger was respectively placed in the purified water and soybean oil on the identical heating platform. Since the sensor exhibited ultra-high sensitivity and the ability to measure the temperature of liquids, this study attempted to use the sensor to detect the reaction process of chemical reactions. The degree of chemical reaction could be evaluated in real time by detecting the change in the temperature of the solution that was attributed to the endothermic heat during the reaction. By complying with the mentioned principle, reactions (e.g., neutralization reactions and most chemical reactions) could be monitored in real time, and the degree of chemical reactions could be revealed from the level of physical information. Figure 5c illustrates the data of dropping of 85 wt.% phosphoric acid (H_3_PO_4_ pH = 1) into the 10 wt.% sodium hydroxide (NaOH pH = 13) solution by using a plastic dropper. In addition, to collect the heat release data of the acid-base neutralization reaction, this study used the flexible temperature sensor attached to the inner wall of the beaker. Besides, the sensor was suggested to exhibit high environmental adaptability, which proved that the sensor could exist in harsh test environments (e.g., acids, lyes and various complex liquid environments). After the acid solution was dropped into the alkali solution, a rapid reaction took place and a large amount of heat was released, causing the temperature to rise significantly. The alkaline solution surrounding the acid solution suffers dilution, slowing the reaction process. However, with the continuous diffusion of molecules, the chemical reaction continued, and the solution continued to be diluted. The cycle of this process led to constant fluctuations in the temperature of the solution in the overall downward trend. In fact, in chemical reactions, most of the combination reaction and acid-base neutralization reaction were accompanied by the absorption and release of heat. The emergence of this sensor could complete the real-time monitoring of chemical reactions which made a non-visualized process become a visible physical parameter. The acquisition of this parameter will greatly improve the intelligent control and detection based on chemical reaction production, saving plenty of time and economic costs. Furthermore, absolute ethyl alcohol acted as a liquid cold source to stimulate a temperature sensor which was placed on the heating table in a thermally stable state. When the absolute ethyl alcohol at ambient temperature dropped to the temperature measurement point of the sensor at 150 °C, the TEMF of the sensor dropped off cliff-like since the absolute ethanol was evaporated. The sensor’s TEMF dropped by 16.9% in 20 ms, which showed the extremely high-temperature sensitivity in Figure 5d. This feature can ensure that the sensor can detect the large-scale temperature changes in the environment in time. For example, it was expected to alert the abnormal temperature change in the process of chemical reaction change in time. Besides, the sensor could detect the extreme temperature changes caused by the destruction of the agricultural greenhouse at night and issue an alarm to remind farmers to make timely countermeasures.

The sensor could perfectly complete the acquisition of the temperature information of the solid and liquid test objects. Next, the temperature detection ability of the sensor for gaseous was tested. A hot blower was adopted as the load to apply temperature, and the air outlet of the blower was respectively facing and flat on the heat sensitive part of the sensor to collected the temperatures. According to the results in Figure 5e, under the continuous action of the hot blower, the thermoelectric signal responded timely as its temperature increased slowly. At the heating phase of the experiment, the test signal fluctuated significantly. However, the TEMF signal became stable immediately after the operation of the hot air blower was terminated. This phenomenon demonstrated that the flexible sensor could always ensure reliable working characteristics, and the fluctuation was attributed to the effect of the convective heat transfer method of the hot air flow. Moreover, the flexible sensor underwent multiple temperature increasing and decreasing tests with a radiant electric heater in Figure 5f. As revealed from Figure 5, with the continuous variation of the thermal radiation intensity, the temperature intensity felt by the sensor was altered constantly, which demonstrated that the sensor exhibits excellent thermal radiation temperature measurement capabilities.

## 5. Conclusions

In brief, a process route for preparing a temperature sensor based on screen printing technology for flexible substrates was proposed. By optimizing the material ratio and post-treatment method, this study determined an In_2_O_3_ thermoelectric film with ultra-high Seebeck coefficient of 175.8 μV/°C. For the ultra-high sensitivity and good temperature retention, the high-performance use of the sensor can be ensured. The prepared flexible sensor was not restricted by the state of the object to be tested and the heat transfer mode that could meet the overall requirements for temperature measurement. The ingenious combination with wearable devices endows flexible temperature sensors with new application potential, so the sensors show the prospect of market applications.

## Figures and Tables

**Table 1:** The parameters of slurries by using screen printing technology.

Group	In_2_O_3_	α-Terpineol	Epoxy Resin	Polyether Amine	Binder:Powder
1	1.400 g	0.150 g	0.140 g	0.077 g	1:10
2	1.400 g	0.150 g	0.280 g	0.154 g	2:10
3	1.400 g	0.150 g	0.420 g	0.231 g	3:10
4	1.400 g	0.150 g	0.560 g	0.308 g	4:10
5	1.400 g	0.150 g	0.700 g	0.385 g	5:10
